# Next-generation sequencing-based tools or nanopore-based tools: which is more suitable for short tandem repeats genotyping of nanopore sequencing?

**DOI:** 10.1093/bioadv/vbaf119

**Published:** 2025-06-12

**Authors:** Wei Han, Xuemei Zhang, Qingzhen Zhang, Zhe Zhou

**Affiliations:** Bioinformatics Center of AMMS, Beijing 100850, P.R. China; Key Laboratory of Epigenetic Regulation and Intervention, Institute of Biophysics, Chinese Academy of Sciences, Beijing 100101, P.R. China; Bioinformatics Center of AMMS, Beijing 100850, P.R. China; Bioinformatics Center of AMMS, Beijing 100850, P.R. China

## Abstract

**Motivation:**

Short tandem repeats (STRs) are widely recognized as critical genetic markers for individual identification. Nanopore sequencing technology holds promise as an effective tool for onsite STR detection owing to its portability. Initially, low sequencing quality led to the development of various genotyping tools specifically tailored for nanopore data. However, recent advancements in nanopore sequencing quality suggest that tools designed for next-generation sequencing (NGS) may be more suitable for analyzing nanopore data than those specifically developed for nanopore sequencing.

**Results:**

We selected two sequencing platforms, MinION Mk1C, and PolySeqOne, to generate sequencing data from 61 unrelated individual samples. Samples were amplified using a custom NanoSTR panel that included 31 autosomal STRs (A-STRs) and 31 Y chromosomal STRs (Y-STRs). Sequencing data were analyzed using four distinct tools: NASTRA, STRspy, STRinNGS, and STRait Razor. Our findings indicated that STRinNGS showed greater accuracy for both A-STRs and Y-STRs, enabling the accurate detection of a broad range of STRs. Compared with STRinNGS, NASTRA exhibited greater STR depth and featured more non-integer stutters. Therefore, in practical applications, STRinNGS demonstrates high reliability in genotyping.

**Availability and implementation:**

NASTRA, STRspy, STRinNGS and STRait Razor, which can be accessed via the following links: https://github.com/renzilin/NASTRA, https://github.com/unique379r/strspy, https://bitbucket.org/rirgabiss/strinngs/src/master, and https://github.com/Ahhgust/STRaitRazor, respectively. The commands during process are provided as requested by the corresponding author.

## 1 Introduction

Short tandem repeats (STRs) are the most commonly used genetic markers for individual identification (Chakraborty *et al.* 1999, Butler 2006). They achieve high identification probability when used with a specific number of STRs, based on statistical principles. STRs are classified into Simple repeats, Compound repeats, and Complex repeats. Simple repeats refer to repeat units that have similar lengths and base compositions. Compound repeats involve two or more motifs at the same locus, with repeat units of similar length. Complex repeats refer to motifs that exhibit both sequence and length variations between alleles at the same locus. STRs are characterized by a high degree of polymorphism and robust single-marker identification power (Gill *et al.* 1994, Tahir *et al.* 2000). Typically, 13 autosomal STR loci suffice for individual identification, 19 loci meet the requirements for paternity testing, and 39 loci are adequate for identifying full sibling relationships (Zhu *et al.* 2015). The Short Tandem Repeat DNA Internet Database (STRBase), maintained by the National Institute of Standards and Technology, catalogs over 70 human STR markers (Ruitberg *et al.* 2001). Many of these markers are widely employed by laboratories around the world to build human DNA databases for public safety purposes.

Capillary electrophoresis (CE) remains the standard method for STR detection. CE operates on the principle that charged molecules migrate through capillaries at rates determined by their size, and STR fragments are analyzed based on their length, as indicated by fluorescent labeling and migration time (Shewale *et al.* 2012). Next-generation sequencing (NGS) has been widely used in forensic science for individual identification, phenotype prediction, ancestry inference, and kinship analysis (Just *et al.* 2015, Oldoni *et al.* 2019, Haas *et al.* 2021). Unlike CE, which detects only length-based polymorphisms (Bredemeyer *et al.* 2022), NGS provides more detailed data by identifying sequence variants of identical lengths (Gettings *et al.* 2016). However, both CE and NGS have common limitations; they tend to be bulky and require recalibration when moved, posing challenges for onsite detection. Recently, several integrated rapid DNA systems, such as Quick TargSeq, ANDE, and RapidHIT 200, have been developed that combine CE with microfluidic technologies to enhance on-site STR detection capabilities. Despite these advancements, significant challenges persist, including limited sample throughput and the inability to obtain comprehensive sequence information (Bowman *et al.* 2022).

The advent of nanopore sequencing technology offers new possibilities for onsite STR detection. Since its introduction in 2014, MinION from Oxford Nanopore Technologies (ONT) has significantly advanced nanopore sequencing research (Deamer *et al.* 2016). This innovative method passes single-stranded DNA through a nanopore-sized protein under a constant voltage and records nucleotide sequences based on changes in ionic current as each nucleotide passes through the pore. By weighing about 87 g, MinION offers sufficient sequencing throughput and real-time data processing capabilities, making it suitable for on-site identification (Quick *et al.* 2014). In 2024, Beijing PolySeq Biotech Co., Ltd launched PolySeqOne, a novel nanopore sequencing device. PolySeqOne provides a high resolution and throughput, making it a strong contender in the field of nanopore sequencing technology (Li *et al.* 2024).

Despite these advantages, nanopore sequencing faces challenges in accurately analyzing STRs, primarily because of its low sequencing quality. Numerous studies have explored the use of MinION for STR genotyping. A recent study by Ren et al. utilized a self-developed tool called NASTRA to genotype data from the R9.4.1 flow cell, achieving accurate genotyping of 18 of 27 loci. When using data from R10.3 flow cell, NASTRA successfully genotyped 16 out of 22 loci (Ren *et al.* 2024).

As nanopore flow cells continue to undergo upgrades, the quality of the sequencing data has improved (Lerminiaux *et al.* 2024). This enhancement raises questions about the suitability of traditionally designed tools for nanopore-based sequencing. Specifically, are the STR typing tools commonly used in NGS better suited for analyzing higher-quality nanopore data? To answer this question, we developed a multiplex amplification kit named NanoSTR that enables the simultaneous detection of 31 autosomal STRs (A-STRs) and 31 Y-chromosome STRs (Y-STRs). The libraries amplified using NanoSTR were sequenced on MGISEQ-2000RS (MGI Tech Co., Ltd), MinION Mk1C, and PolySeqOne. For our analysis, we selected four tools: NASTRA (Ren *et al.* 2024) and STRspy (Hall *et al.* 2022), designed for nanopore sequencing, and STRinNGS (Jønck *et al.* 2020) and STRait Razor (Woerner *et al.* 2017), widely applicable tools originally designed for NGS. We performed a comprehensive comparative evaluation of the STR typing capabilities of the two nanopore sequencing devices by assessing their detection rates, accuracy rates, and sequencing depths. Finally, we summarized the characteristics of error-prone STRs to provide guidance for subsequent algorithm optimization. The results demonstrated that STRinNGS can accurately genotype a greater number of STRs, making it a more suitable tool on the nanopore platform.

## 2 Methods

### 2.1 Locus selection and primer design

All the study samples were amplified using a custom-developed NanoSTR panel. STR loci were selected using commercially available kits, including the STRtyper-32G PCR Amplification Kit and SureID PathFinder Plus Kit (Health Gene Technologies, Ningbo, China). The NanoSTR primer set was designed using Primer Premier 5.0 and MFEprimer (Qu *et al.* 2009) to ensure primer specificity, optimal length, and suitable annealing temperatures. The customized panel included 31 A-STRs and 31 Y-STRs. Detailed information is provided in Table 1, available as supplementary data at *Bioinformatics Advances* online.

### 2.2 Sample collection and quantification

Blood card samples (Nuhigh Biotechnologies, Suzhou, China) were collected from 60 unrelated Han Chinese individuals (43 males and 17 females). Standard DNA 9948 (AGCU ScienTech Incorporation, Wuxi, China) was diluted to a concentration of 1 ng/µl and used as a male sample. DNA concentrations were determined using a Qubit 3.0 Fluorometer (Thermo Fisher Scientific, Waltham, MA, USA).

### 2.3 PCR amplification and library construction

PCR amplification involves two stages. The purpose of the PCR1 step was to amplify target fragments. The 30 µl reaction mix included 3.5 µl Enhancer buffer NB (1 N), 2.5 µl Enhancer buffer M, 5 µl primer pool (containing all STR and InDel primers listed in Table 2, available as supplementary data at *Bioinformatics Advances* online), 10 µl EM808 polymerase mixture, and 9 µl DNA (or nuclease-free water for blood card samples). The PCR program included 95°C for 3.5 min; 24 cycles of 98°C for 20 s, 60°C for 4 min; and 72°C for 5 min. After magnetic bead purification, a 30 µl PCR2 was performed to enrich the target fragments. The reaction mixture contained 13.5 µl of the purified product, 2.5 µl Enhancer buffer M, 2 µl nuclease-free water, 2 µl UDI primer, and 10 µl EM808 polymerase mixture. The PCR thermal cycler settings for this stage were: 95°C for 3 min 30 s; nine cycles of 98°C for 20 s, 58°C for 1 min, and 72°C for 30 s; and 72°C for 5 min. The blood card sample requires a puncher to obtain a 2 mm diameter disc from the blood card. This disc is then mixed with the PCR1 reaction system, allowing for immediate PCR amplification. After the second purification, the library concentration was measured using 1 µl of the sample on a Qubit 3.0 Fluorometer. The quality, purity, and integrity of the library were assessed using the Bioptic Qsep 100 Bio-Fragment Analyzer.

### 2.4 Nanopore sequencing

DNA library aliquots from NanoSTR were used for library preparation with the SQK-NBD114.96 kit (ONT) and the NEBNext Library Prep Kit (New England Biolabs, Beijing, China). To enhance sequencing efficiency, bovine serum albumin (Invitrogen, Carlsbad, CA, USA) was added to the flow cell priming mix. Prepared flow cells were sequenced using MinION Mk1C (ONT), and raw read data were base-called with Dorado 7.2.13 in super-accurate mode.

For the PolySeqOne platform (POLYSEQ), product aliquots were prepared for library construction. The PY-DLB101 kit was used for end modification and barcode ligation, and the PY-BLP101 kit was used for adapter ligation. The magnetic beads in the kit were used for DNA purification at each step. The prepared libraries were then loaded onto flow cells and sequenced on PolySeqOne using the default settings in the PolyseqCtrl software.

### 2.5 Next-generation sequencing and capillary electrophoresis

NanoSTR product aliquots underwent rolling circle amplification (RCA) to generate sufficient DNA nanoballs (DNBs) for sequencing on the MGISEQ-2000RS platform. STR typing of FASTQ files was performed using STRait Razor 3.0. For CE, A-STRs were amplified using the STRtyper-32G PCR Amplification Kit, and Y-STRs were amplified using the SureID PathFinder Plus Kit. PCR products were analyzed using a 3730XL DNA Analyzer (Thermo Fisher Scientific). Data analysis was performed using GeneMapper ID-X v1.6.

### 2.6 STR typing based on nanopore sequencing

The STR typing of FASTQ files generated by ONT and POLYSEQ was performed using four tools: NASTRA, STRspy, STRinNGS, and STRait Razor.

For NASTRA, nanopore sequencing data were first aligned to the human reference genome (GRCh37, hg19) using Minimap2 (Li 2018), then converted to “bam” format with SAMtools (v1.13) (Li *et al.* 2009). NASTRA structures STR genotyping based on configuration files outlining the repeat information of the loci. The original configuration file contained 54 STR sites. We expanded this list by adding information on 8 A-STRs (D22S1045, D8S1132, D6S477, D4S2366, D3S3045, D19S253, D15S659, and D10S1435) and 13 Y-STRs (DYS385, DYS393, DYS456, DYS458, DYF387S1, DYS389I, DYS389II, DYF404S1, DYS444, DYS557, DYS593, DYS596, and DYS645) that were not included. NASTRA output includes genotype information along with supporting read depths. Quality control metrics for genotyped STR loci include “interpretation threshold,” “imbalance,” and “nocalling.”

STRinNGS was configured to contain information on 62 STR loci and direct STR typing was conducted using FASTQ data. Genotypes, sequencing depth, and comments for each locus were exported in the CSV format. Comments included terms such as “Allele not defined”, “Unexpected sequence detected”, and “Too few reads”, among others.

For STRspy, we configured a database of 62 STR alleles, and then retrieved the genotypes and sequencing depth based on the typing results. STRait Razor was configured with information on 62 STR loci based on the flanking sequences of the STRs.

The genotyping standards for STRait Razor, STRinNGS, and STRspy were set to their default values. For NASTRA, due to varying supporting read number ratios (SNR) across different loci, we had set the SNR to 0.35 for the newly configured loci, which was consistent with the majority of loci in NASTRA.

The stutter filter ratio in MGISEQ was set to 0.15 for DYS385, and 0.1 for the other loci; for the ONT platform, it was set to 0.3; no filtering ratio was set for the POLYSEQ platform.

### 2.7 Statistical analysis

Linear regression was used to analyze the correlation between the mapping rates of the reference genome data from the two sequencing devices. Data visualization and analysis were performed using GraphPad Prism 9.5 and Microsoft Excel (v2021). STR nomenclature adheres to the recommendations of the International Society for Forensic Genetics (Gettings *et al.* 2024).

## 3 Results

### 3.1 Run summary

**Figure 1.**
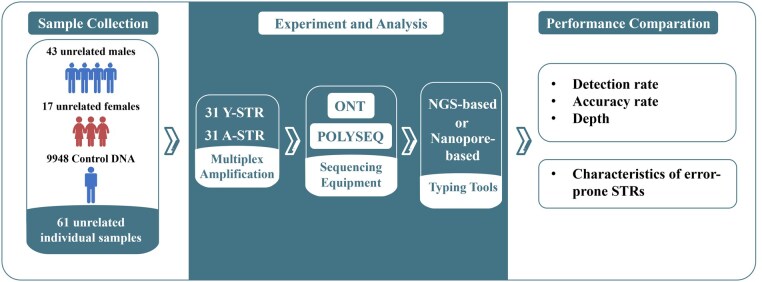
Overview of the study design.

The experimental workflow was illustrated in Fig. 1. DNA samples were collected from 61 unrelated individuals and amplified using NanoSTR including 31 A-STRs and 31 Y-STRs. Sequencing was conducted on both MinION Mk1C and PolySeqOne platforms to compare the performance of 4 STR typing tools.

Four sequencing runs were conducted on MinION Mk1C, processing 15, 15, 15, and 16 samples, respectively. Each run lasted 72 h and generated a total of 80.28 million reads, with an average quality score of 16.5. Each sample yielded an average of 1.32 ± 0.42M reads and an average read length of 281 ± 14 bp. After alignment using minimap2, 97.27 ± 2.80% of the reads were mapped to the human reference genome GRCh38 (G38).

Similarly, four sequencing runs were performed on the PolySeqOne, processing 1, 24, 24, and 12 samples for 2, 42, 44, and 47 h, respectively. These runs produced a total of 81.95 million reads with an average quality score of 14.5. Each sample generated an average of 1.34 ± 0.36M reads with an average read length of 320 ± 55 bp. After alignment using minimap2, 96.73 ± 1.34% of the reads were mapped to G38.

These data indicated that while PolySeqOne (POLYSEQ) exhibited slightly lower performance than the MinION (ONT) platform in terms of sequencing quality, read length uniformity, and the proportion of reads aligned to G38, there was high consistency between the two platforms when examining the proportion of reads aligned to G38 (Pearson correlation coefficient *R^2^* = 0.685; Fig. 1, available as supplementary data at *Bioinformatics Advances* online). The lowest mapping rates for both platforms were observed for sample M8, at 93.23% for POLYSEQ and 89.78% for ONT. The detailed information is summarized in Table 3, available as supplementary data at *Bioinformatics Advances* online.

In conclusion, both the ONT and POLYSEQ platforms met the requirements for sequencing short amplicons and provided adequate sequencing capacity. Sufficient reads were generated for downstream analysis by optimizing the number of samples per run.

### 3.2 Preliminary comparison of NGS-based and nanopore-based tools

We first validated the NGS typing results of NanoSTR against those from CE. Except for DYS448 (1) and DYF387S1 (2), which showed locus drop-outs (LDO), all other STR typing results were consistent with CE-based types, confirming the reliability of MGI typing data as a reference for subsequent comparisons with nanopore sequencing. In the following study, we compared the genotyping capabilities of different tools using metrics such as detection rates and accuracy rates. Detection rates refer to the ratio of detected loci to expected loci. According to the loci in NanoSTR, each sample should detect 31 A-STR loci, and 31 A-STR loci should be detected across all samples. When a tool detected only 30 loci, achieving a detection rate of 96.77% (30/31). Accuracy rates refer to the ratio of loci correctly typed to all detected loci. A tool detected 30 STR loci and correctly typed 25 loci, achieving an overall accuracy rate of 83.34% (25/30).

Among the forensic STR typing tools designed for nanopore sequencing, NASTRA and STRspy performed better typing ability. On ONT platform, NASTRA accurately genotyped 41 STRs at 100% accuracy, while STRspy accurately genotyped 34 STRs. On POLYSEQ platform, NASTRA accurately genotyped 22 STRs at 100% accuracy, while STRspy accurately genotyped 21 STRs. It was evident that NASTRA consistently detected more STRs correctly than STRspy on both ONT and POLYSEQ platform, which was consistent with the findings in previous literature (Ren *et al.* 2024).

Additionally, we found that NGS tools were also applicable to nanopore sequencing. Therefore, we selected two commonly used STR typing tools for NGS—STRinNGS and STRait Razor—and performed genotyping on both ONT and POLYSEQ platforms. On ONT platform, STRinNGS accurately genotyped 55 STRs at 100% accuracy, while STRait Razor accurately genotyped 52 STRs. On POLYSEQ platform, STRinNGS accurately genotyped 30 STRs at 100% accuracy, while STRait Razor accurately genotyped 28 STRs. The results show that STRinNGS consistently detects more STRs correctly compared to STRait Razor.

In conclusion, among the forensic STR typing tools designed for nanopore sequencing, NASTRA exhibits higher accuracy. Among the NGS-based STR typing tools, STRinNGS shows superior accuracy. Therefore, a detailed comparison of the performance of NASTRA and STRinNGS will be provided to identify the optimal forensic STR typing tool for nanopore sequencing.

### 3.3 Comparison of STR genotyping using NASTRA and STRinNGS

#### 3.3.1 ONT

**Figure 2.**
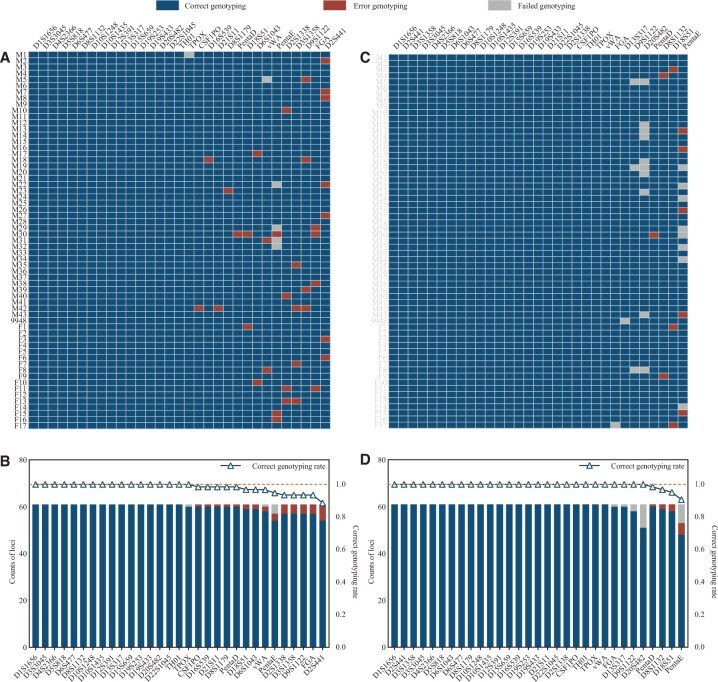
A-STR typing results for NASTRA and STRinNGS using ONT platform. (A) A raster plot of NASTRA typing results. (B) A stacked bar chart displaying correct, error, and failed typing across loci in NASTRA, supplemented by a line graph for accuracy rates. (C) A raster plot of STRinNGS typing outcomes. (D) A stacked bar chart illustrating correct, incorrect, and failed typing for each locus in STRinNGS, with a line graph for accuracy rates. Blue indicates correct genotyping, red indiactes error genotyping, and gray indicates failed genotyping. Loci are arranged in the descending order of accuracy rates.

We evaluated STR typing performance on ONT sequencing data using two tools, NASTRA and STRinNGS. The genotypes of the A-STRs are summarized in Fig. 2. NASTRA detected 1885 loci, achieving a detection rate of 99.68%. Six loci were not detected: three were due to “Interpretation threshold” and three to “Imbalanced.” NASTRA correctly typed 1848 loci, with an overall rate of 98.04%, achieving 100% accuracy for 17 loci. STRinNGS detected 1868 loci, with a detection rate of 98.78%. It missed 23 loci due to “Too few reads.” STRinNGS correctly typed 1857 loci, achieving an overall rate of 99.41% and obtained 100% accuracy for 27 loci. Despite its higher detection rate, NASTRA showed slightly lower accuracy than STRinNGS, which correctly typed more loci. This discrepancy may be attributed to NASTRA’s superior integration of an STR structure-aware tool, which enhances detection, but can introduce challenges in distinguishing certain alleles. Among the 61 samples, we observed 30 isometric alleles (which shared the same size but differed in sequence), including 11 with motif variations and 19 with single-base mutations. Both NASTRA and STRinNGS accurately typed all eleven motif variations. However, of the 19 single-base mutations, only one was correctly typed by NASTRA, whereas STRinNGS accurately typed all mutations. This highlights NASTRA’s limitations in handling single nucleotide polymorphisms (SNPs) or slight base differences between alleles.

For Y-STRs, NASTRA detected 1267 alleles with a detection rate of 92.89% (Fig. 2, available as supplementary data at *Bioinformatics Advances* online). It failed to detect 97 loci, primarily DYS385 (44), DYS596 (36), and DYF387S1 (15). Issues arose from algorithmic limitations for DYS385 and DYS596 and “Interpretation threshold” or “Nocalling” for DYF387S1, which was also observed in MGISEQ. NASTRA correctly typed 1145 alleles, achieving an accuracy rate of 90.37%, with 100% accuracy for 25 Y-STRs. Meanwhile, STRinNGS identified 1344 alleles with a detection rate of 98.53%, missing only 20 loci due to “Too few reads.” It accurately detected 1296 alleles, achieving an accuracy rate of 96.21% with perfect accuracy for 28 Y-STRs. Compared with STRinNGS, NASTRA had significantly lower detection and accuracy rates, partly because of loci with multiple alleles, such as DYS385, DYF387S1, and DYF404S1. These loci have multiple positions in the reference genome, leading to allele dropouts in NASTRA, which aligns FASTQ files to the reference genome. Excluding these three problematic loci, NASTRA achieved a detection rate of 97.00% and an accuracy rate of 95.65%, which were still lower than those of STRinNGS. Notably, both software tools failed to type DYS389II; however, STRait Razor 3.0 could correct this error, suggesting that the issue was algorithmic rather than due to sequencing quality.

#### 3.3.2 POLYSEQ

**Figure 3.**
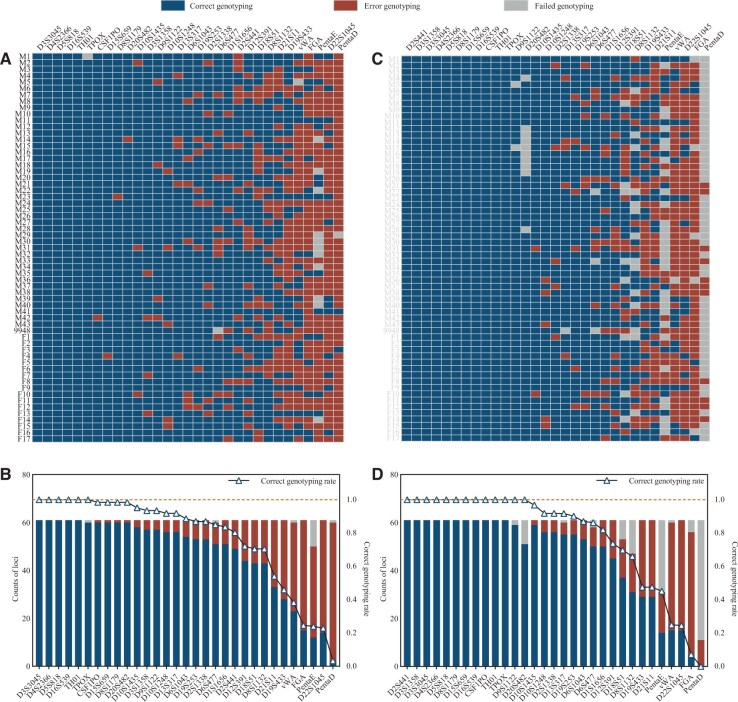
A-STR typing results for NASTRA and STRinNGS using POLYSEQ platform. (A) A raster plot of NASTRA typing results. (B) A stacked bar chart displaying correct, error, and failed typing across loci in NASTRA, supplemented by a line graph for accuracy rates. (C) A raster plot of STRinNGS typing outcomes. (D) A stacked bar chart illustrating correct, incorrect, and failed typing for each locus in STRinNGS, with a line graph for accuracy rates. Blue indicates correct genotyping, red indicates error genotyping, and gray indicates failed genotyping. Loci are arranged in the descending order of accuracy rates.

The average quality score for POLYSEQ was 14.5, compared to 16.5 ONT, indicating a lower overall accuracy, as illustrated in Fig. 3. For A-STRs, NASTRA detected 1876 alleles with a detection rate of 99.21%. 15 loci were undetected, primarily in PentaE (12), with causes including “Imbalance” (5) and “Interpretation” (7). NASTRA correctly typed 1457 alleles with an accuracy of 77.67%. STRinNGS detected 1767 alleles, with a detection rate of 93.44%. All undetected loci were due to insufficient read depth. It correctly typed 1434 alleles, with an accuracy of 81.15%. While NASTRA outperformed in terms of detection rate, STRinNGS outperformed in terms of accuracy. Similar to the ONT data, NASTRA faced challenges in accurately typing isometric alleles in the POLYSEQ data. NASTRA correctly typed 9/11 motif variations, whereas STRinNGS correctly typed 10. For 19 single-base mutations, NASTRA correctly typed only 1 mutation, whereas STRinNGS correctly typed 17. NASTRA correctly typed 6 A-STRs, whereas STRinNGS correctly typed 13.

For Y-STRs, NASTRA detected 1271 alleles, with a detection rate of 93.18% (Fig. 3, available as supplementary data at *Bioinformatics Advances* online). The undetected loci were primarily DYS385 (44), DYS596 (33), and DYF387S1 (15). It correctly typed 985 alleles with 77.50% accuracy, including 16 Y-STRs that were accurately genotyped. STRinNGS detected 1307 alleles with a detection rate of 95.82%, missing 23 loci due to “Too few reads.” It correctly detected 1030 alleles with an accuracy of 78.81%, including 17 accurately genotyped Y-STRs. Similar to the ONT data, NASTRA struggled to accurately type multiple-allele loci, such as DYS385, DYF387S1, and DYF404S1, in the POLYSEQ data. It also had difficulty detecting DYS596, correctly typing only 11 of 44. STRinNGS faced challenges with DYF387S1, correctly typing only 8 of 44, likely due to the lower sequencing quality of POLYSEQ, which hinders accurate alignment.

**Figure 4.**
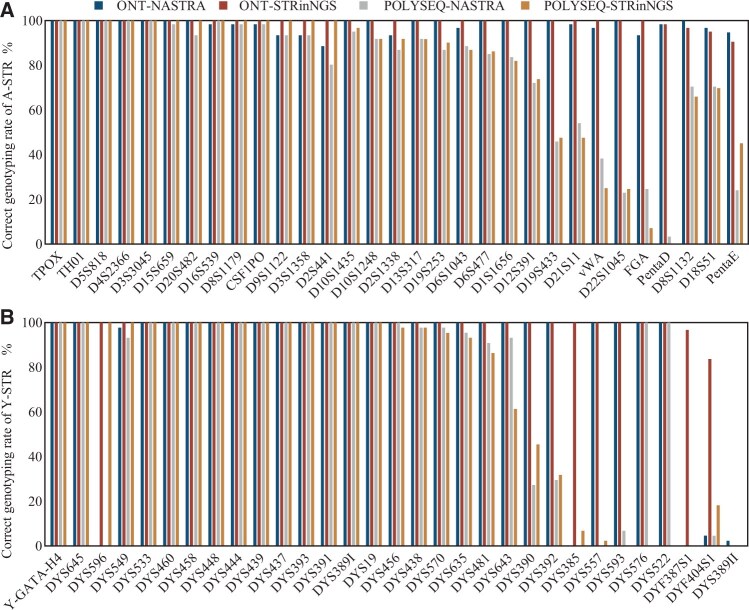
Comparative genotyping accuracy between NASTRA and STRinNGS using ONT and POLYSEQ platform. (A) Bar chart for A-STR correct typing rates across four groups; (B) Bar chart for Y-STR correct typing rates across four groups. Blue denotes NASTRA typing ONT data, red denotes STRinNGS typing ONT data, gray denotes NASTRA typing POLYSEQ data, and orange denotes STRinNGS typing POLYSEQ data. Loci are organized using ONT-STRinNGS typing accuracy from high to low.

In summary, with nanopore platforms, NASTRA performed better in A-STR detection than STRinNGS, but was weaker for Y-STRs. NASTRA’s accuracy was lower than that of STRinNGS, primarily because of challenges with isometric alleles. In ONT, NASTRA correctly detected 17 A-STRs, whereas STRinNGS detected 27. On POLYSEQ, NASTRA correctly typed six A-STRs, whereas STRinNGS correctly typed 13. For Y-STRs, NASTRA correctly typed 25 in ONT and 16 in POLYSEQ, which were both lower than the 28 and 17 typed by STRinNGS, respectively (Fig. 4). These findings demonstrate that STRinNGS has advantages in terms of typing accuracy, leading to more reliable outcomes than NASTRA.

### 3.4 Sequencing depths comparison

**Figure 5.**
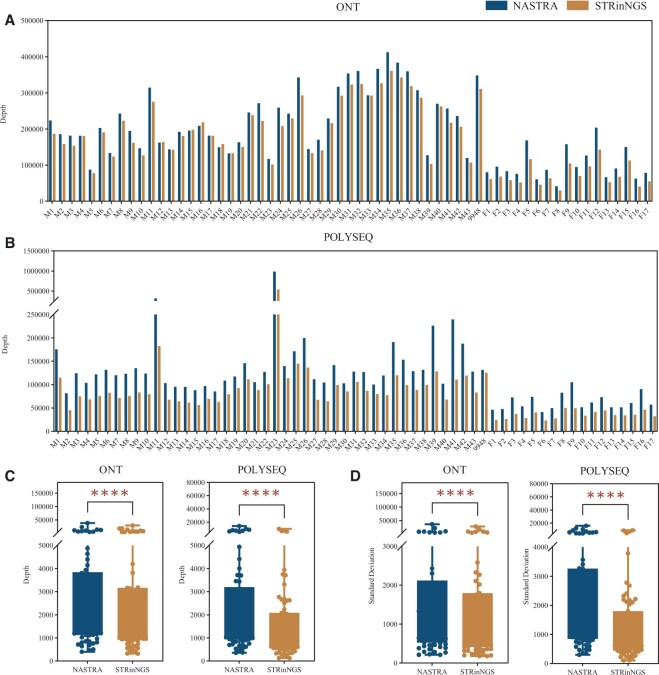
Depths for NASTRA and STRinNGS using ONT and POLYSEQ platform. (A) Bar chart depicting depths of each sample in ONT. (B) Grouped bar chart depicting depths of each sample in POLYSEQ. (C) Box plot of average depth for each locus, with points indicating actual average depth. (D) Box plot of standard deviations for depth at each locus, with points showing actual standard deviations. Blue indicates NASTRA and orange indicates STRinNGS.

The depth refers to the number of reads mapped to a particular locus in a single sequencing run. Figure 5A illustrates the sequencing depth of each sample on the ONT platform, revealing a strong correlation between NASTRA and STRinNGS. Except for samples M16 and M18, all samples exhibited greater depths with NASTRA than with STRinNGS. This highlighted NASTRA’s superior performance in processing samples with lower data volumes. In POLYSEQ (Fig. 5B), advantage of NASTRA was even more pronounced, showing higher depths for all samples compared to STRinNGS. For the depth of each locus on the ONT, NASTRA demonstrated higher average depths than STRinNGS, except for a few Y loci (DYS385, DYS596, DYS481, DYF387S1, and DYF404S1) with software design issues (detailed in Section 3.2.1). In POLYSEQ, only DYS385 and DYS596 showed lower average depths in NASTRA, and all other loci surpassed those of STRinNGS.

A box plot (Fig. 5C) comparing the average depth across all loci showed that NASTRA achieved significantly higher mean depths than STRinNGS on both the ONT and POLYSEQ platforms, with a more pronounced difference observed in POLYSEQ. The standard deviation of the depths for each locus was also calculated (Fig. 5D), indicating that NASTRA had a significantly higher standard deviation than STRinNGS, which was particularly evident in POLYSEQ. This suggests that while NASTRA achieves higher depths, it does so with greater fluctuations, particularly when dealing with lower-quality data.

### 3.5 Characteristics of error-prone STRs on nanopore devices

Using the PolySeqOne platform, which was known for its relatively high error rate, we analyzed genotyping errors in NASTRA and STRinNGS. In NASTRA, 13 of 62 STRs showed accuracies below 30% (Fig. 4), including 4 A-STRs and 9 Y-STRs. Among these, four were simple STRs, three were compound, and six were complex (Table 4, available as supplementary data at *Bioinformatics Advances* online). Fisher’s exact test revealed a significant difference in genotyping accuracy between simple and complex STR types (*P *= 0.0178), but not between simple and compound types. Of the 13 STRs with accuracies below 30%, 12 contained homopolymers [repeated bases such as the (AAAGA)n structure in PentaD, which features four consecutive adenines]. In contrast, only 13 of the remaining 49 STRs contained homopolymers. Fisher’s exact test revealed a significant correlation between homopolymers and lower accuracy (*P *≤ 0.0001).

In STRinNGS, 10 of the 62 STRs had accuracies below 30%, comprising 4 A-STRs and 6 Y-STRs, with two simple, two compound, and six complex. Fisher’s exact test suggested that STR errors may be correlated with complex STR types (*P* = 0.0033). Among the 10 STRs with accuracies below 30%, nine contained homopolymers, whereas 16 of the remaining 52 STRs contained homopolymers. Fisher’s exact test confirmed a significant correlation (*P *= 0.0007).

Due to the relatively low error rate of the ONT platform, we focused on loci with accuracies below 95% for further analysis. In NASTRA, 11 out of 62 STRs had accuracies of less than 95%, with four being simple, three compound, and four complex (Table 5, available as supplementary data at *Bioinformatics Advances* online), and 8 out of 11 contained homopolymers. Fisher’s exact test indicated that higher STR error rates were unrelated to STR type but were associated with the presence of homopolymers (*P *= 0.0211). In STRinNGS, only four STRs showed accuracies below 95%, all of which contained homopolymers.

Thus, on the POLYSEQ platform, complex STRs were more susceptible to genotyping errors, and homopolymers significantly affected the genotyping accuracy. No correlation was found between the STR class and accuracy on the ONT platform; however, homopolymers consistently affected STR typing. This aligns with the findings of Yang et al. on the QNome (Yang *et al.* 2024), indicating a common issue across nanopore sequencing platforms. These findings underscore the importance of considering the STR class and homopolymer content when interpreting nanopore sequencing data.

**Figure 6.**
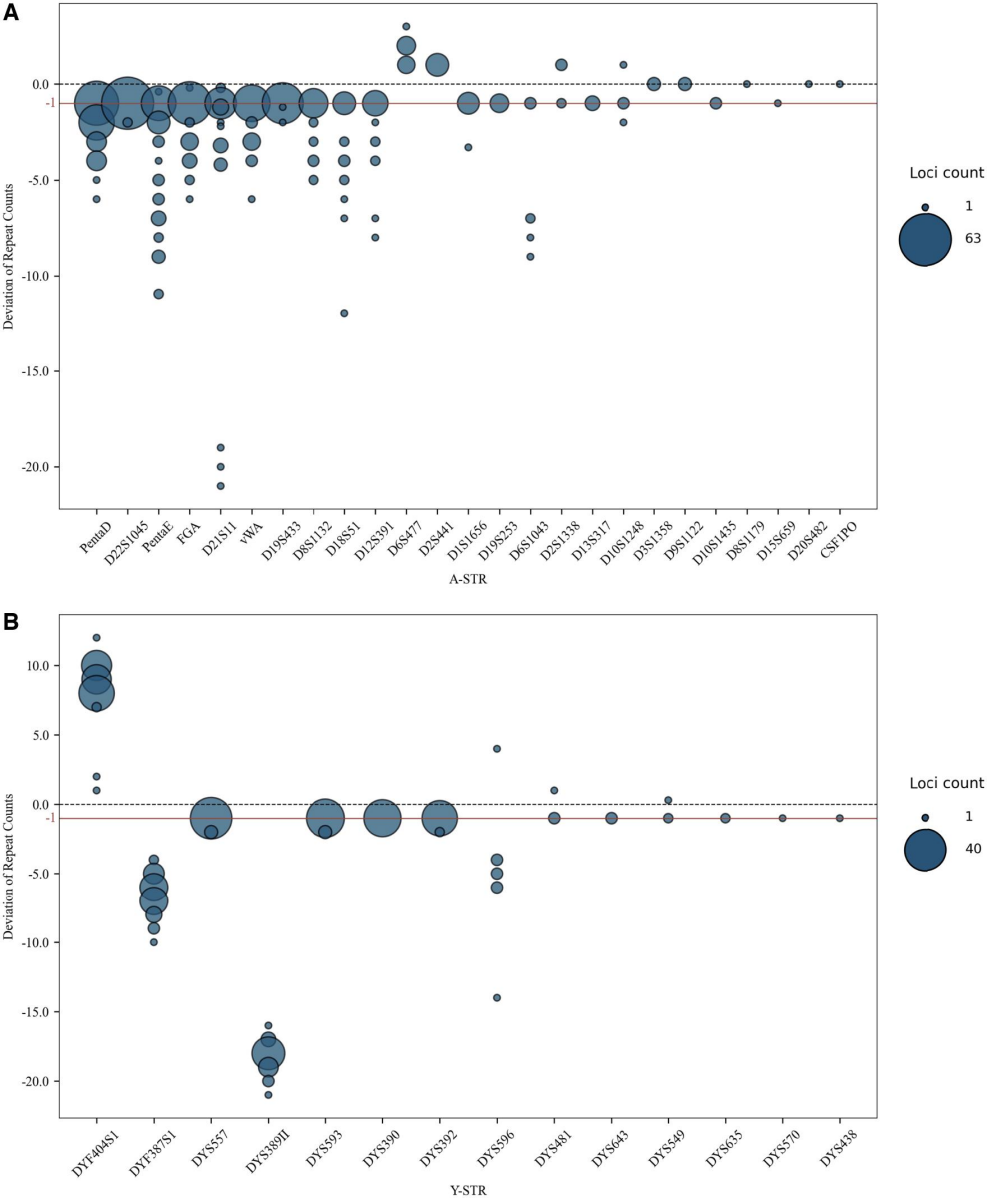
Deviations of repeat unit counts from the true alleles based on POLYSEQ platform using NASTRA. (A) Deviations of A-STR repeat unit counts from the true alleles for 61 samples. Each allele is listed separately. (B) Deviations of Y-STR repeat unit counts from the true alleles for 44 male samples.

We subsequently analyzed stutter in POLYSEQ. STRs typically consist of consecutive nucleotide repeats, such as the repeat motif [TCTA]n for D2S441. The genotypes for 9948 are [TCTA]11 and [TCTA]12. The genotype of [TCTA]10 indicates a ‘-1’ deviation stutter, where one repeat unit is missing compared to the correct genotype, while [TCTA]13 indicates a ‘+1’ deviation stutter, with one additional repeat unit compared to the correct genotype. For NASTRA, 66.08% (378/572) of the errors in A-STRs and 42.94% (149/347) in Y-STRs were one-repeat deviations from the benchmark. Additionally, 355 and 147 “−1” deviations were found among all one-repeat errors, respectively (Fig. 6). Under STRinNGS, 63.32% (290/458) in A-STRs and 69.76% (173/248) in Y-STRs were one-repeat deviations. Among all one-repeat errors, we found 285 and 170 “−1” deviations, respectively (Fig. 4, available as supplementary data at *Bioinformatics Advances* online). This indicates that “−1” stutter peaks are the most common, highlighting POLYSEQ’s sequencing bias. Furthermore, NASTRA exhibited a 2.5% (23/919) rate of non-integer stutter peaks, which was significantly lower than that of STRinNGS (13.74%; 97/706). This relates to NASTRA’s process of inferring repeat structures, as it prioritizes the detection of potential motifs (e.g., AAAG and AGAA) during genotyping, leading to integer outcomes.

## 4 Conclusion

Nanopore sequencing technology represents a substantial advancement in genomics, transforming traditional DNA sequencing into long-read analysis and real-time data acquisition. However, the accurate identification of bases remains challenging because multiple nucleotides pass through the nanopore protein simultaneously (Wang *et al.* 2021). Previous research has focused on enhancing STR detection accuracy, leading to advanced tools, such as DeepRepeat (Fang *et al.* 2022), STRspy (Hall *et al.* 2022), NanoSTR (Lang *et al.* 2023), and the newly launched NASTRA (Ren *et al.* 2024). These advancements have significantly broadened the applications of nanopore sequencing in forensics. When the sequencing quality surpasses a certain threshold, we observed that forensic STR tools commonly used in NGS also provide accurate typing of nanopore data.

NASTRA is divided into two main modules: read clustering and repeat-structure inference. Read clustering identifies candidate alleles by filtering minor noise, whereas a recursive repeat search algorithm reconstructs the repeat structures of alleles using known motifs from reference databases. This approach is crucial when nanopore sequencing is less accurate but may introduce issues as sequencing quality improves. We evaluated a total of 61 samples using ONT and POLYSEQ platforms. Although NASTRA demonstrated superior detection performance for A-STRs compared to STRinNGS, STRinNGS exhibited higher accuracy for both A-STRs and Y-STRs, enabling it to correctly detect a greater number of STRs. In some cases, although STRinNGS may fail in STR genotyping, the detected STRs are generally more reliable.

In addition to evaluating the detection and accuracy rates, the depth variations of the two tools were compared. Our analysis revealed that, aside from a few loci, NASTRA generally produced higher depths than STRinNGS. However, the NASTRA depths showed greater fluctuations than those of STRinNGS. Subsequent error type analysis identified a common issue across both platform: loci containing homopolymers are difficult to type accurately, and “−1” motif stutter peaks are more prone to be introduced. This finding provides a clear direction for future algorithm optimization.

Our study has several limitations as follows: (ⅰ) Sample collection was limited to Chinese Han individuals, potentially missing some alleles. (ⅱ) Thresholds for NASTRA’s initial STRs were optimized; however, those for the supplementary STRs in our experiment were relatively arbitrary, which may have affected the accuracy of NASTRA.

In conclusion, the combination of ONT and STRinNGS accurately typed 27 A-STRs, exceeding the 18 A-STRs reported in the recent ONT literature (Ren *et al.* 2024). The combination of POLYSEQ and STRinNGS accurately typed 13 A-STRs, outperforming QNome in recent studies (Yang *et al.* 2024) and making it the most suitable nanopore equipment for STR typing after ONT. However, the quality of nanopore sequencing is inferior to that of NGS. The accurate typing of STRs with numerous homopolymers remains challenging, underscoring the need for advancements in base-calling and genotyping tools.

## Supplementary Material

vbaf119_Supplementary_Data

## Data Availability

Raw data of standard samples have been deposited to the National Center for Biotechnology Information (NCBI) under the BioProject number PRJNA1256619.
