# Supplementary material for: Next-generation sequencing-based tools or nanopore-based tools: which is more suitable for short tandem repeats genotyping of nanopore sequencing?
Source: Bioinform Adv. 2025 Jun 12;5(1):vbaf119. doi: 10.1093/bioadv/vbaf119 (PMC12167636; doi:10.1093/bioadv/vbaf119)

**Supplementary Figure. S1.** Correlation analysis of the mapping rate of sequencing data of every sample to the reference genome (GRCh38) on ONT and POLYSEQ platform.
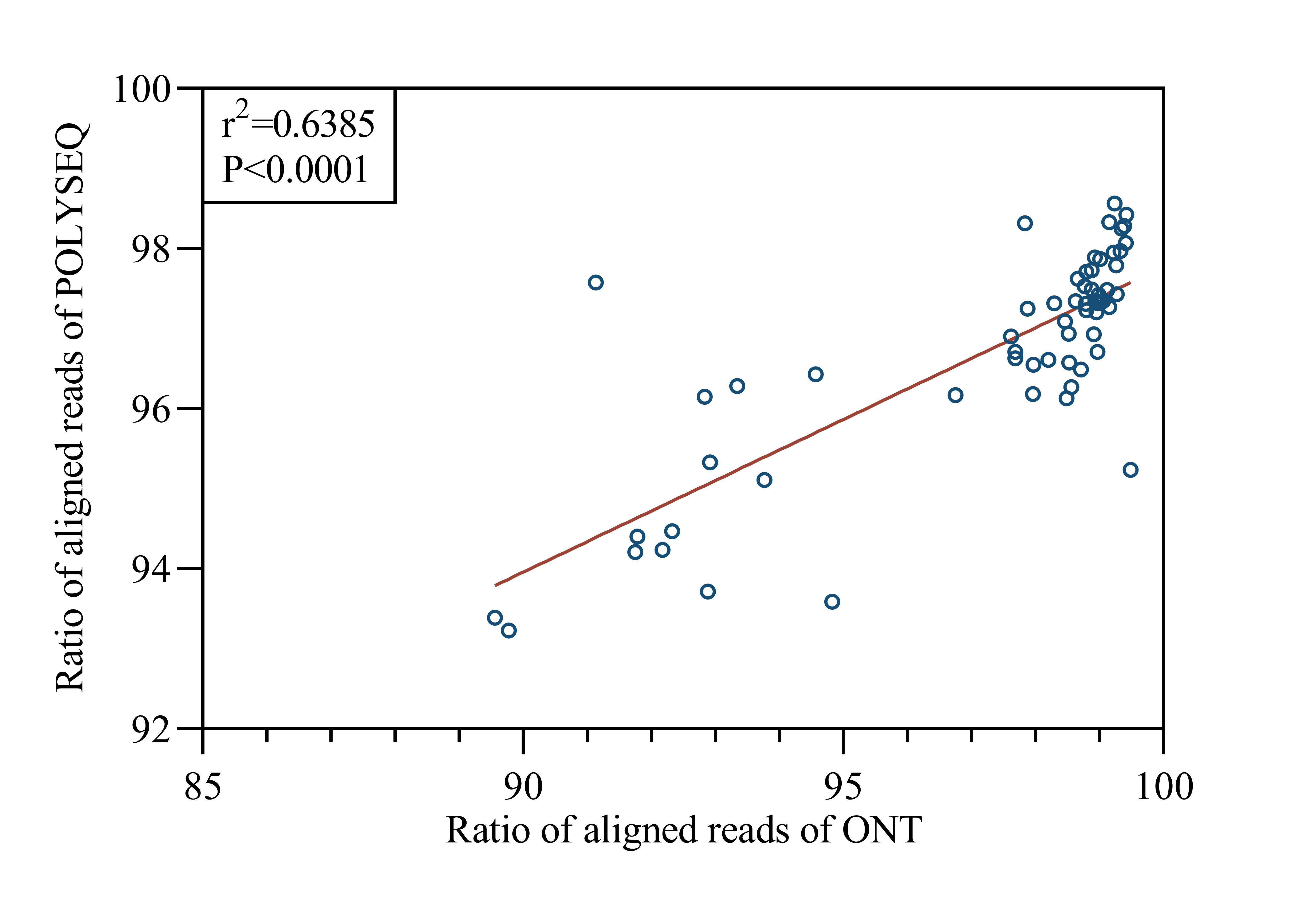


**Supplementary Figure. S2.** Y-STR typing results for NASTRA and STRinNGS using ONT platform. A) A raster plot of NASTRA typing results. B) A stacked bar chart displaying correct, error, and failed typing across loci in NASTRA, supplemented by a line graph for accuracy rates. C) A raster plot of STRinNGS typing outcomes. D) A stacked bar chart illustrating correct, incorrect, and failed typing for each locus in STRinNGS, with a line graph for accuracy rates. Blue indicates correct genotyping, red indiactes error genotyping, and gray indicates failed genotyping. Loci are arranged in the descending order of accuracy rates.





**Supplementary Figure. S3.** The Y-STR typing results for NASTRA and STRinNGS using POLYSEQ platform. A) A raster plot of NASTRA typing results. B) A stacked bar chart displaying correct, error, and failed typing across loci in NASTRA, supplemented by a line graph for accuracy rates. C) A raster plot of STRinNGS typing outcomes. D) A stacked bar chart illustrating correct, incorrect, and failed typing for each locus in STRinNGS, with a line graph for accuracy rates. Blue indicates correct genotyping, red indicates error genotyping, and gray indicates failed genotyping. Loci are arranged in the descending order of accuracy rates.





**Supplementary Figure. S4.** Deviations of repeat unit counts from the true alleles based on POLYSEQ equipment using STRinNGS. (A) Deviations of A-STR repeat unit counts from the true alleles for 61 samples. Each allele is listed separately. (B) Deviations of Y-STR repeat unit counts from the true alleles for 44 male samples.


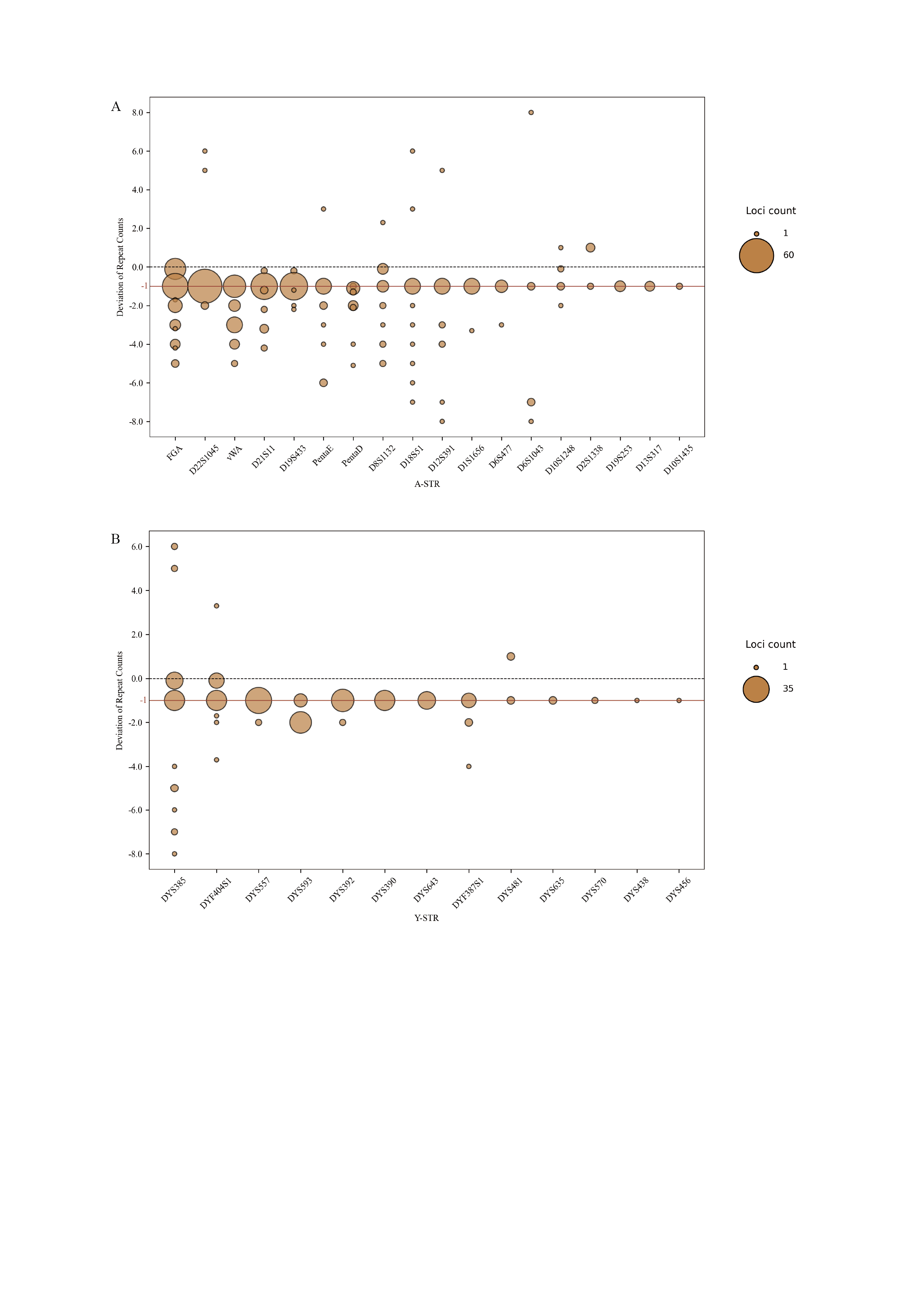

Supplement: vbaf119_Supplementary_Data [file vbaf119_supplementary_data.zip › Supplementary Figure.docx]
